# Dressing-induced allergic contact dermatitis in total joint arthroplasty

**DOI:** 10.1007/s00264-025-06715-w

**Published:** 2025-12-05

**Authors:** Farouk Khury, Garrett Ruff, Sophia Antonioli, Daniel Sherwood, Ran Schwarzkopf, Joshua Rozell

**Affiliations:** 1 Technion Israel Institute of Technology, Haifa, Israel; 2 Division of Orthopedic Surgery, Rambam Health Care Campus, Haifa, Israel; 3 Department of Orthopedic Surgery, New York University Langone Medical Center, New York, United States

**Keywords:** Allergic contact dermatitis, Arthroplasty dermatitis, THA dermatitis, TKA dermatitis, Wound dressing dermatitis, Wound dressing THA, Wound dressing TKA

## Abstract

**Purpose:**

To investigate the incidence and risk factors for dressing-induced allergic contact dermatitis (DIACD) following total hip and knee arthroplasty (THA and TKA, respectively) across different dressings and sealants.

**Methods:**

A retrospective review was conducted of patients who underwent primary, elective THA or TKA between 2019 and 2024 with ≥ 90 days of follow-up. Incidences of DIACD were identified by reviewing medical records for “allergy” diagnoses and use of antihistamines or corticosteroids within 30 days postoperatively. Patient characteristics, prior exposure, treatment, dressing type, and allergy history were analyzed.

**Results:**

A total of 61 (0.3%) of the 23,396 investigated patients developed a DIACD on average 12.2 ± 7.3 days postoperatively. Overall, 41% had a preoperative allergy (excluding seasonal), and 55.7% were treated with topical or low-dose oral antihistamines and corticosteroids. The majority (41%) of the DIACD involved mesh-adhesive dressings, and a liquid skin adhesive (2-octyl cyanoacrylate) was also used in 41% of cases, often in combination with the primary dressing. Of the 61 DIACD patients, 24 (39.3%) had previously undergone THA or TKA, and nearly half of these (*n* = 11, 45.8%) had been exposed to the same dressing without prior occurrence of DIACD. DIACD patients were significantly more likely to have undergone TKA (73.8 vs. 58.3%, *p* = 0.015) and to have never smoked (75.4 vs. 58.4%, *p* = 0.014). The effect sizes of these findings were negligible (Cramer’s V = 0.016 and 0.019, respectively).

**Conclusions:**

The incidence of DIACD following joint arthroplasty is low (0.3%) but remains a frustrating complication, primarily occurring two weeks postoperatively, with mesh-adhesive dressings most frequently implicated. Patients with prior exposure to dressings, those undergoing TKA, and non-smokers are at higher risk. Identifying at-risk patients can guide dressing selection and application.

## Introduction

Postoperative sterile bandages and sealants provide a barrier for healing tissue and are important tools to achieving successful surgical outcomes following total hip arthroplasty (THA) and total knee arthroplasty (TKA) [1]. Despite advancements in low-profile dressing design, such as those integrating tissue adhesives, polymer mesh backing, and silver-impregnated hydrofiber layers, wound healing challenges remain a persistent concern following surgery [1, 2]. Among these, dressing-induced allergic contact dermatitis (DIACD) has emerged in several publications [3–9] as an important yet often underrecognized condition warranting further investigation.

Beyond THA and TKA, reports of allergic contact dermatitis (ACD), particularly related to mesh-adhesive dressings, are documented in literature on shoulder arthroplasty [10], anterior cervical spine fusion [11], abdominal approach for lumbar spine fusion [12], and plastic surgery [13, 14]. These works describe the possibility of a delayed-type IV hypersensitivity reaction requiring a sensitization phase, during which an individual who has never been previously exposed to a specific dressing is first exposed to a chemical allergen, and a subsequent exposure then triggers the clinical manifestation of dermatitis five to 14 days following initial contact [15].

Although ACD symptoms are mostly mild, presenting as a macular or maculopapular pruritus [3–8], more severe reactions can involve systemic symptoms and full-body rash. At worst, some reactions require debridement and skin grafting [15, 16]. Such severe outcomes, combined with the potential for DIACD to be misdiagnosed as cellulitis or other surgical site infection, underscore the importance of early identification, proper dressing selection, and effective wound management.

Furthermore, although the incidence of DIACD after THA and TKA is generally reported between 0.5% and 2.8% [5, 17, 18], and up to 8.1% [19] in patients with prior exposure with mesh-glue dressings, underreporting remains common in the context of total joint arthroplasty (TJA). This study aims to (1) investigate the incidence and risk factors for DIACD following TJA, (2) analyze the clinical characteristics of DIACD, including the time to presentation and types of dressings most frequently implicated, and (3) highlight the difference in clinical course from surgical site infection, thus guiding appropriate treatment through a retrospective analysis of patients who underwent primary THA or TKA at a high-volume academic hospital and subsequently developed DIACD. We hypothesize that the incidence of DIACD is low but statistically significantly associated with specific patient characteristics (such as prior surgery) and dressing types (namely mesh-adhesive dressings).

## Materials and methods

### Study design

Following Institutional Review Board (IRB) approval, a retrospective review was conducted of patients who underwent primary THA or TKA between March 2019 and July 2024 at a single, urban, academic hospital. Primary query yielded 26,873 patients. Allergic reactions to wound dressings were identified through a review of medical records for International Classification of Diseases (ICD) codes related to “allergy” and documented prescription or administration orders for any form of antihistamines or corticosteroids within 30 days following surgery. Patients were included in the study if they were older than 18 years of age, had undergone primary THA or TKA and indicated for primary or secondary osteoarthritis, osteonecrosis of the femoral head or femoral neck fracture non-union, had complete operative reports, home visiting nursing records (which often provide the first documentation of localized rash/pruritus in the early postoperative period), and follow-up visits detailing the progress of an allergic reaction to a dressing, and where the primary clinical assessment noted findings consistent with contact dermatitis (e.g., pruritus, macular/papular or small vesicular rash in the same distribution as the location of the dressing) and lacked systemic signs of infection (fever, chills, elevated inflammatory markers). Patients were excluded if they had any prior soft tissue, bone, or joint infection, any previous operation on the same surgical site including revision procedures, any prior immunosuppressant treatment, or any data lacking information regarding the dressing used. Following exclusion, the final study cohort consisted of 23,396 patients, 61 of which had an allergic reaction to one of the dressings. This group was referred to as “DIACD”. Due to the retrospective nature of the patient identification query, all 61 DIACD patients necessarily had a documented prescription or administration order for either antihistamines, corticosteroids or a combination of these medications. All DIACD patients underwent removal of the dressing. None of the DIACD patients were treated with any form of antibiotic treatment.

### Data collection

Our institution’s electronic medical record (EMR) database (Epic Caboodle. Version 15; Verona, Wisconsin, USA) was utilized to collect the “allergic” patients’ demographics (e.g. gender, age, body mass index (BMI), race, smoking status, American Society of Anesthesiologists (ASA) scores, type of arthroplasty performed (hip or knee)), past medical, allergy, surgical, social histories, data regarding the type of wound dressing implicated, and documentation of any allergic reaction to it during the outpatient clinic visits. Seasonal allergies were excluded from the analysis due to their low clinical correlation to contact dermatitis and potential confounding of the data. Data regarding the type of wound dressing implicated were categorized as mesh-adhesive (Dermabond Prineo^®^), simple (gauze/absorbent pad with surgical tape), hydrofiber (Aquacel^®^), and negative-pressure wound therapy (PICO™ or Prevena™). All dressings were applied in the operating room by the surgical team according to the respective manufacturer’s standard protocol. Quality metrics were also assessed, including time until diagnosis of an allergic reaction, method of treatment used (antihistamines, corticosteroids, or a combination of multiple treatments), 90-day emergency department (ED) visits and readmission rate, reoperations and revisions.

### Data analyses

Baseline characteristics of patients were represented as count with percentages for categorical variables and means ± standard deviations for continuous variables. In addition to descriptive statistics, the statistical model consisted of (1) *chi*-squared (x^2^) test for assessing differences in categorical variables between DIACD and non-DIACD cohorts, and (2) Student’s t-test for comparing continuous variables between the groups. Multivariate analysis was not performed due to the low absolute number of DIACD events (*n* = 61), which limits the power to control for confounding variables. In cases where a significant finding was observed, effect size (ES) was calculated for Cohen’s d (numerous parameters: 0.2–0.5 small, 0.5–0.8 medium, > 0.8 large) and Cramer’s V (categorical parameters: 0.1–0.3 small, 0.3–0.5 medium, > 0.5 large). Significance was set as a *p*-value less than 0.05. All statistical analyses were conducted using SPSS Statistics (Version 28; IBM Corporation, Armonk, New York, USA).

## Results

### Patient demographics

Of the 23,396 investigated cases, sixty-one patients (0.3%) (32.8% males and 67.2% female), with a mean age of 67.5 years (range, 34 to 93 years), were identified as having a DIACD following THA (26.2%) or TKA (73.8%) with a mean follow-up time of 1.2 ± 1.3 years. The majority of both groups were non-smokers. These two observations were found to be significantly different, as DIACD patients were significantly more likely to have never smoked (75.4 vs. 58.4%, *p* = 0.005) and to have undergone TKA rather than THA (73.8 vs. 58.3%, *p* = 0.040). DIACD patients were significantly more likely to have ASA score III than non-DIACD patients (45.9 vs. 35.8%, *p* = 0.030). Although significant, the effect sizes of these findings were negligible (Cramer’s V = 0.019 for smoking status, 0.015 for ASA score, and 0.016 for type of procedure). Student’s t-test demonstrated no significant different in age (67.5 vs. 66.3 years, *p* = 0.270) and BMI (31.2 vs. 31.3 kg/m^2^, *p* = 0.980) between the groups (Table 1).

**Table 1:** Patient demographics and features

Parameter	Allergic group (*n* = 61)	Non-allergic group (*n* = 23,335)	*p*-value *(ES)*
**Mean age at surgery** [range], years	67.5 [34–93]	66.3 [14–97]	0.270
**Sex**, *n* (%)			
Male	20 (32.8)	8,846 (37.9)	0.340
Female	41 (67.2)	14,489 (62.1)	
**Mean BMI** [range], (kg/m^2^)	31.2 [20.3–46.9]	31.3 [13.8–74.4]	0.980
**Smoking status**, *n* (%)			
Never	46 (75.4)	13,623 (58.4)	0.005* (0.019)
Former	11 (18.0)	8,371 (35.9)	
Current	4 (6.6)	1,341 (5.7)	
**ASA score**, *n* (%)			
I	0 (0.0)	978 (4.2)	0.003* (0.015)
II	33 (54.1)	13,750 (58.9)	
III	28 (45.9)	8,365 (35.8)	
IV	0 (0.0)	238 (1.0)	
**Procedure performed**, *n* (%)			
TKA	45 (73.8)	13,614 (58.3)	0.040* (0.016)
THA	16 (26.2)	9,721 (41.7)	
**Mean follow-up time** ± SD, years	1.2 ± 1.3		

SD, standard deviation; *n*, number; %, percentage; ES, effect size; BMI, body-mass index; ASA, American society of Anesthesiologists

### Prior exposures to wound dressings

In terms of allergic history and prior exposure, 41% of the DIACD patients (*n* = 25) had a documented preoperative allergy history to either antibiotics (*n* = 14, 56%), food (*n* = 4, 16%), latex (*n* = 4, 16%), or other medication (*n* = 3, 12%), excluding seasonal allergies. Among the 61 patients who experienced DIACD, 24 (39.3%) had a history of a previous arthroplasty where similar wound dressings were applied. Additionally, of these 24 patients, 11 (45.8%) were exposed to the same type of dressing used in the prior procedure. In this subgroup, the prior exposure predominantly involved 2-octyl cyanoacrylate or mesh-adhesive dressings (*n* = 8, 72.7%), followed by simple (*n* = 2, 18.2%) and negative-pressure wound therapy (*n* = 1, 9.1%) dressings. The remaining 13 patients (54.2%) had been exposed to a different dressing type previously, mainly standard gauze (*n* = 11, 84.6%). Furthermore, 19 of the 24 patients (79.2%) had a liquid skin adhesive used during their prior procedure, with a simple dressing (*n* = 9, 47.4%), a mesh-adhesive dressing (*n* = 8, 42.1%), hydrofiber (*n* = 1, 5.3%), and NPWT (*n* = 1, 5.3%). Regardless, none of these patients reported any allergic reactions following their previous procedure (Table 2).

**Table 2:** Description of allergy history and prior arthroplasty in the allergic group

Parameter	Allergic group (*n* = 61)
^§^**Prior allergy diagnosis**, *n* (%)	25 (40.9)
Antibiotics	14 (56.0)
Nutrition	4 (16.0)
Latex	4 (16.0)
Other medication	3 (12.0)
Prior arthroplasty, *n* (%)	24 (39.3)
Exposure to same dressing	11 (45.8)
Mesh-adhesive	8 (72.7)
Simple	2 (18.2)
NWPT	1 (9.1)
Exposure to different dressing	13 (54.2)
Simple	11 (84.6)
NWPT	1 (7.7)
Hydrofiber	1 (7.7)
Exposure to skin adhesive	19 (79.2)
With mesh-adhesive dressing	8 (42.1)
With simple dressing	9 (47.4)
With hydrofiber	1 (5.3)
With NPWT	1 (5.3)

*n*, Number; %, Percentage; ^§^, diagnosis of a previous known allergy to a specific medication, nutrition or latex; NPWT, negative-pressure wound therapy (PICO or Prevena)

### Characteristics of DIACD

The allergic reactions were attributed to a variety of wound dressings, with mesh-adhesive dressings being the most common (*n* = 25, 41%), followed by simple dressings (gauze-based) (*n* = 17, 27.9%), hydrofiber (*n* = 11, 18%), and negative-pressure wound therapy dressings being the least common (*n* = 8, 13.1%). In addition to the primary dressing, a liquid skin adhesive was used for skin closure in 25 (41%) of the DIACD cases. Among these, the adhesive was used in conjunction with a mesh-adhesive dressing in 12 cases (48.0%), a simple dressing in five cases (20%), a hydrofiber dressing in four cases (16%), and NPWT in four cases (16%). The majority of the reactions presented clinically as erythema/eczema (*n* = 52, 85.2%), redness (*n* = 51, 83.6%), pruritus (*n* = 35, 57.4%), papules (*n* = 10, 16.4%), swelling (*n* = 8, 13.1%), blisters (*n* = 4, 6.5%), and urticarial rash (*n* = 3, 4.9%) (Table 3). There were no reported cases of severe systemic allergic reaction.

**Table 3:** Descriptive statistics of the allergic reactions and dressing types used

Parameter	Allergic group (*n* = 61)
**Dressing type**, *n* (%)	
Mesh-adhesive	25 (41.0)
TKA	21 (84.0)
THA	4 (16.0)
Simple	17 (27.9)
TKA	10 (58.8)
THA	7 (41.2)
Hydrofiber	11 (18.0)
TKA	8 (72.7)
THA	3 (27.3)
NPWT	8 (13.1)
TKA	6 (75.0)
THA	2 (25.0)
**Use of skin adhesive**, *n* (%)	25 (41.0)
With mesh-adhesive dressing	12 (48.0)
With simple dressing	5 (20.0)
With hydrofiber	4 (16.0)
With NPWT	4 (16.0)
**Mean time until diagnosis** ± SD, (days)	12.2 ± 7.3
**Time until diagnosis within 30 POD**, *n* (%)	55 (90.1)
Same day	4 (7.2)
Within 3 days	8 (14.5)
Within 7 days	13 (23.6)
Within 14 days	33 (60.0)
**Clinical presentation**, *n* (%)	
Erythema/eczema	52 (85.2)
Redness	51 (83.6)
Pruritus	35 (57.4)
Papules	10 (16.4)
Swelling	8 (13.1)
Blisters	4 (6.5)
Urticarial rash	3 (4.9)
**Prescribed medication**, *n* (%)	34 (55.7)
Antihistamines	24 (70.5)
Corticosteroid tablets	21 (61.7)
Corticosteroid creme	6 (17.6)
Combination	13 (38.2)
**90-day ED visit**, *n* (%)	10 (16.4)
Due to allergic reaction	6 (60.0)
Due to other medical cause	4 (40.0)

*n*, Number; %, Percentage; NPWT, negative-pressure wound therapy (PICO or Prevena); POD, postoperative day; ED, emergency department

Patients who suffered from DIACD presented at a mean 12.2 ± 7.3 days postoperatively. Nearly all cases (*n* = 55, 90.1%) were diagnosed within 30 days. The majority of these cases (60%) were diagnosed within 14 days of surgery, followed by 23.6% who were diagnosed within seven days of surgery, 14.5% who were diagnosed within three days of surgery, and 7.2% who were diagnosed within the same day of surgery. 36.4% of diagnoses were determined between the first and second week postoperatively. Treatment for these reactions involved antihistamines (*n* = 24, 70.5%), oral corticosteroids (*n* = 21, 61.7%), or a combination of antihistamines and oral/topical corticosteroids (*n* = 13, 38.2%). The average time from initiation of treatment until documented clinical resolution of symptoms (defined as complete resolution of rash and pruritus) was 7.4 ± 4.1 days. Description of the antihistamine and corticosteroid treatments including dosages are detailed in Table 4. Of the entire cohort, 16.4% visited the ED within 90 days of their procedure, with 60% of these visits directly related to their allergic reaction. The first half of these DIACD-related 90-day ED visits were prescribed oral corticosteroids with doses ranging from four to 20 mg, and the second half were prescribed antihistamines. There were no documented readmissions, reoperations or revision surgeries during the investigated time frame.

**Table 4:** Treatment regimens prescribed for DIACD patients

Medication, *n* (%)		DIACD patients who were prescribed medication (*n* = 34)
**Antihistamines**	**First-generation**	
	Diphenhydramine	32 (94.1)
	12.5 mg/5 ml	2 (6.2)
	25 mg	24 (75)
	50 mg	6 (18.8)
	Hydroxyzine	20 (58.8)
	10 mg	2 (10)
	25 mg	11 (55)
	50 mg	7 (35)
	**Second-generation**	
	Loratadine	9 (26.5)
	10 mg	9 (100)
	Cetirizine	4 (11.8)
	10 mg	4 (100)
	**Third-generation**	
	Fexofenadine	7 (20.6)
	60 mg	4 (57.1)
	180 mg	3 (42.9)
	Desloratadine	2 (5.9)
	5 mg	2 (100)
**Corticosteroids**	**Methylprednisolone**	15 (44.1)
	4 mg	15 (100)
	**Prednisone**	6 (17.7)
	5 mg	1 (16.7)
	10 mg	1 (16.7)
	20 mg	4 (66.7)

*n*, Number; %, percentage

## Discussion

This retrospective study provides the largest known analysis of DIACD following THA and TKA, offering valuable insights into its incidence, risk factors, and clinical characteristics. Although the incidence of DIACD in our large cohort was relatively low (0.3%) compared to previous reports [5, 15, 17–19], the clinical implications for affected patients remain significant, as DIACD can result in concerning manifestations, potential misdiagnosis as surgical site infection, and subsequently trigger an unnecessary diagnostic workup. While this likely delayed-type IV hypersensitivity reaction predominantly involved mesh-adhesives, a finding that is consistent with prior studies [3–7], analysis revealed an interesting, significantly higher prevalence of DIACD following TKA in non-smokers. These findings highlight the importance of identifying patients at risk to inform the selection and application of suitable wound dressings. TKA incisions are under more tension compared to hip incisions, leading to increase shear forces on the wound and the bandage itself during range of motion activities. Furthermore, delaying proper diagnosis and treatment could lead to secondary complications possible due to bacterial penetration of the vulnerable skin [20].

In recent years, various wound closure and dressing techniques [2, 21–23] have been introduced, aiming to create optimal conditions for skin healing and prevent complications. Factors such as permeability and absorptive capacity, risk of infection, and cost should be considered when deciding on which dressing type to apply [2]. Among the frequently recommended dressings [1, 2], hydrofibers (both non- and silver-impregnated) have been reported to have a lower infection and ACD rate than simple dressings. Despite the apparent superiority of hydrofibers, the literature contains conflicting evidence regarding their effectiveness, as they were also associated with more episodes of delayed wound healing and reoperations when compared with mesh-dressings in previous publications [24, 25].

Primarily due to their ease of application and the ability to bathe immediately without heightened risks of dehiscence or infection [26–29], mesh-adhesive dressings became widely popular. Furthermore, these dressings were reported [24] to enhance strength and provide tension-sharing properties compared to simple dressings and were recommended for use by the 2024 International Delphi Study on Wound Closure and Incision Management in THA [30] and TKA [31]. Despite these strengths, this study found that most of the allergic reactions (41%) had mesh-adhesives as their dressing. Similar observations of mesh-adhesive-induced ACD have been published in various surgical fields [3–16, 26, 32] with incidence rates up to 7%. For 41% of the patients in our cohort, a liquid skin adhesive (2-octyl cyanoacrylate) alone was applied before the primary dressing. The combination of a liquid adhesive and outer bandage may confound the causative allergen. There also may be a dose dependent effect of the liquid, as the volume used in conjunction with the mesh along with the surface area of the mesh itself is likely more than if the liquid is used alone. It is important to highlight that while the Food and Drug Administration (FDA) [33] approves mesh-adhesives for surgical incisions in a low-tension area, it warns against its use in high friction areas and in patients with specific comorbidities such as peripheral vascular disease, clotting disorders, insulin-dependent diabetes mellitus. Notwithstanding, this study as well as current literature did not investigate the possible correlation of these comorbidities to DIACD. This should be investigated in future research and wound dressings ought to be applied following manufacturer instructions in order to avoid undesirable outcomes [34]. Furthermore, mesh-adhesives might contain acrylic resin monomers that are known to induce skin sensitization as they are commonly found in various materials, including nail cosmetics, dental products and printing inks [35]. Therefore, patients with possible prior exposure to these substances might benefit from preoperative patch testing or use of other dressing than mesh-adhesives.

Delayed-type IV hypersensitivity reactions characteristically consist of a sensitization stage during the initial contact with a chemical allergen, followed by a subsequent exposure stage that leads to dermatitis symptoms, which appear ten to 14 days after the initial contact or within hours or a few days during repeated exposure [36]. While 60% of the patients in this study were diagnosed with DIACD within 14 days, and 36.4% within seven to 14 days as to be expected in a delayed hypersensitivity reaction, 14.5% and 7.2% of the diagnoses were within seven and three days, respectively (Table 3). In these reactions, we believe that the initial sensitization is due to an immunological cross-reaction between the dressing and other allergens, particularly acrylate compounds found in cosmetic products [37–39]. This complexity is further emphasized by our finding that nearly half (45.8%) of patients with a history of a previous surgery were exposed to the same type of dressing, and nearly 80% were exposed to liquid skin adhesive, without any prior allergic reaction. The development of DIACD on subsequent exposure, despite previous tolerance, suggests a nuanced sensitization process that may be triggered by factors beyond simple repeat contact, such as changes in immune response or variations in allergen concentration. Such complex cases highlight the importance of considering preoperative dermatologic consult or testing when planning high-risk dressing application, particularly in patients with a suspicion of possible acrylates exposure prior to TJA [40, 41].

The treatments chosen (topical/oral corticosteroids and antihistamines) reflect the standard management protocol for a type IV delayed hypersensitivity reaction [42], aiming to reduce inflammation and pruritus. The strength of the topical steroid or addition of oral steroids was dictated by the severity of the skin reaction. The average time to resolution of 7.4 days further supports the effectiveness of this standard symptomatic approach. These findings are highly relevant as the clinical presentation of DIACD can often mimic early-stage cellulitis or a superficial surgical site infection, frequently leading to unnecessary and often broad-spectrum antibiotic prescriptions. The successful resolution of all 61 cases with only local wound care, antihistamines, and corticosteroids supports the use of a non-antibiotic regimen for clinically confirmed DIACD, while maintaining a low threshold for investigation if signs of systemic infection are present.

DIACD were significantly more common in TKAs than THAs. Although this finding had a rather negligible effect size (Cramer’s V = 0.016), this has been theorized by Chalmers et al. [15], who despite having reported a higher DIACD incidence rate than this study (0.5% vs. 0.3%), hypothesized that skin adhesive dressings on extensor surfaces are tensioned to the point of microfracturing and releasing more allergen and causing more irritation which in turn starts the delayed-type hypersensitivity reaction. While this hypothesis seems to be interesting, we assume that simply due to the frequent knee joint movement and flexion compared to the hip, the dressing shifts and causes friction which increases the risk of reaction, a concern consistent with the FDA’s recommendation [33] to avoid using this type of dressing in high-friction areas. Furthermore, the skin around the knee is thinner and more sensitive than the hip joint, making it more prone to irritation from adhesives. In addition, TKA often involves a larger incision and more tissue manipulation which might lead to more damage and higher risk of reaction. While theoretically all types of dressings might cause reactions, the vast majority (84%) of the DIACDs in TKAs were using mesh-adhesives (Table 3). Given these findings, we advise that patients with a history of adhesive reactions or atopic conditions undergo preoperative allergy testing, an in-office 2-octyl cyanoacrylate challenge, or simply use of a potentially less allergenic dressing in TKA. For patients identified as high-risk for a skin reaction, surgeons should consider alternatives to mesh-adhesive dressings, such as negative pressure wound therapy, silk fibroin dressings, or non-adhesive gauze with paper tape.

While neither age nor BMI exhibited any correlation to the development of an allergic reaction, which is in contrast to a previous publication by Coles et al. [4], non-smokers were found to have statistically significantly more episodes of DIACD when compared to current or former smokers (75.4% vs. 58.4% non-smokers in the non-allergic cohort, *p* = 0.005). This observation may be due to the known immunosuppressive effect of nicotine, which dampens immune responses and decreases Th_2_-related cytokine activity associated with type IV hypersensitivity reactions [43, 44]. Nicotine has shown to inhibit eosinophil migration and suppress the production of Th2 chemokines and cytokines, which play a critical role in allergic inflammation [43]. Additionally, longitudinal studies have also established that smokers have a lower prevalence of allergic sensitization compared to non-smokers, suggesting that smoking alters immune responsiveness in a way that reduces the likelihood of allergic reactions [45]. These findings highlight the complex immunomodulatory effects of smoking, which may explain the observed differences in allergic reactions. Nonetheless, it is important to highlight that the effect size of this observation was negligible, perhaps making its clinical relevance uncertain.

### Limitations

Even though this analysis demonstrated several noteworthy findings, it is not without limitations. This study was limited by its retrospective nature; data regarding patients’ comorbidities was not analyzed. This might be of clinical interest, as the FDA has cautioned against use of mesh-adhesives in patients with specific conditions [33]. Furthermore, the data query of patients’ EMRs might have missed cases of allergic reactions due to patients self-treating using over-the-counter medication without seeking medical attention or not having a formal “allergy” diagnosis. This may also explain the lower incidence rate of DIACD in our cohort compared to previous literature. We did not assess DIACD cases that were not treated medically, and thus these cases might have been missed. In addition, data on whether a dermatological consultation or testing was performed was not available. Furthermore, even though the analysis of prior arthroplasty confirmed whether the type of dressing was similar, the EMR data did not allow for a detailed analysis of the specific components or brand variations of the dressings used in the prior procedure versus the current procedure, which may be a limitation in fully understanding the sensitization process. Additionally, while this study reported on the entire cohort that underwent TJA, including those that did and didn’t develop DIACD, it lacked an appropriate control group, making it challenging to isolate the impact of the dressing alone on outcomes, as other factors may be confounding. This limitation diminishes the strength of the conclusions and highlights the need for future comparative studies. Furthermore, the potential bias in dressing selection is a limitation, influenced by factors like surgeon’s familiarity, hospital preferences, and ease of application. As this study represents a retrospective census of our entire cohort to capture all 61 cases due to the low incidence of DIACD, a formal a priori power analysis was not applicable. However, we acknowledge that the low absolute number of DIACD events (*n* = 61) means the statistical power is limited for detecting subtle or complex associations. Furthermore, while we documented the dressing type for all 61 DIACD patients, we did not track dressing type for the entire non-DIACD cohort. Therefore, the dressing type frequencies presented in the Results section represent the distribution within the affected patients, not a comparative risk or incidence between different dressing types. These factors may not align with key aspects of wound management, potentially affecting the generalizability of the findings. The variability in clinical presentation and treatment of DIACD among patients could introduce variability in outcomes, affecting the reliability of the findings. In addition, while the cohort in this study is, to the best of our knowledge, the largest in current literature to be investigated on this topic, the findings demonstrated may not represent the overall incidence of DIACD, particularly since there are a variety of reported rates [3–16, 26, 32]. Furthermore, this study cannot draw any conclusions about the association between specific dressing types and infection, a topic that has been previously investigated by authors from our institution [1], as there were no cases of infection in this study. Nonetheless, in contrast to previous publications [4, 15], we advise against antibiotic treatment in cases of ACD (Table 4).

## Conclusions

In conclusion, while the incidence of DIACD after TJA in this study cohort is relatively low, this side effect can be troublesome, typically occurring around two weeks (12.2 days) postoperatively, which aligns with both the delayed Type IV hypersensitivity reaction timeline and the timing of initial follow-up, with mesh-adhesive dressings most often implicated. All patients were successfully treated (defined as complete clinical resolution of rash and pruritus) using either antihistamines, corticosteroids or a combination of these medications. No cases of DIACD resulted in infection, and careful practice of antibiotic stewardship in these cases is important. Patients with prior dressing exposure, TKA, or no smoking history are at higher risk, likely due to type IV hypersensitivity, skin tension from knee motion, and heightened immune responses, respectively. These findings highlight the importance of identifying at-risk patients, diagnosing and treating properly, as delays in addressing clinical symptoms could lead to secondary complications.

## Data Availability

No datasets were generated or analysed during the current study.
